# Use of Integral Forage Palm Flour as an Innovative Ingredient in New Fettuccine-Type Pasta: Thermomechanical and Technological Properties, and Sensory Acceptance

**DOI:** 10.3390/foods13172683

**Published:** 2024-08-26

**Authors:** Luiz Eliel Pinheiro da Silva, Sander Rodrigues Moreira, Nathalia de Andrade Neves, Etiene Valéria de Aguiar, Vanessa Dias Capriles, Tatiana Nunes Amaral, Marcio Schmiele

**Affiliations:** 1 Institute of Science and Technology, Federal University of Jequitinhonha and Mucuri Valleys (UFVJM), Diamantina 39100-000, Brazil; luiz.eliel@ufvjm.edu.br (L.E.P.d.S.) sander.moreira@ufvjm.edu.br (S.R.M.); nathalia.neves@ict.ufvjm.edu.br (N.d.A.N.); tatiana.amaral@ict.ufvjm.edu.br (T.N.A.); 2 Laboratory of Food Technology and Nutrition, Department of Biosciences, Institute of Health and Society, Campus Baixada Santista, Federal University of São Paulo (UNIFESP), Santos 11015-020, Brazil; etiene.aguiar@unifesp.br (E.V.d.A.); vanessa.capriles@unifesp.br (V.D.C.)

**Keywords:** formulation, microwave dehydration, phenolic compounds, protein network, sustainability, technological properties, texture

## Abstract

Dehydrated integral forage palm cladode flour (FPF) presents a promising nutritional and functional approach to enriching fettuccine-type pasta. This study investigated the use of microwave-dehydrated FPF (at 810 W) as a partial wheat flour substitute (0, 5, 10, 15, and 20% *w*/*w*) in fresh and dry fettuccine-type pasta. The thermomechanical properties of flour blends and the technological and sensory attributes of the resulting pasta were evaluated. FPF displayed a high protein (15.80%), mineral (15.13%), dietary fiber (67.35%), and total soluble phenolic compound (251 mg EAG·100 g^−1^) content. While water absorption (~58%) and dough stability remained consistent across formulations, a decrease in maximum torque during heating was observed (*p* < 0.05). Fettuccine-type pasta containing 10% FPF exhibited an acceptable optimal cooking time, solid loss, weight gain, and textural properties for both fresh and dry pasta. Sensory evaluation revealed acceptability above 63% for pasta with 10% FPF, with a slight preference for the fresh version. Fresh pasta flavored with garlic and extra virgin olive oil (garlic and oil pasta) achieved a sensory acceptance rate of 79.67%. These findings demonstrate the potential of FPF for fettuccine-type pasta production, contributing desirable technological characteristics and achieving acceptable sensory profiles.

## 1. Introduction

*Opuntia ficus-indica* L. Miller, commonly known as forage palm, is a Mexican cactus with remarkable adaptability. It thrives in arid and semi-arid regions characterized by high temperatures and scarce water resources. This resilience makes it a crucial source of water and forage for livestock in drought-prone areas [1,2]. Recognizing its importance, the Food and Agriculture Organization of the United Nations (FAO) highlights forage palm’s potential for development in these regions. Cultivation of forage palm by small-scale producers using simple and appropriate technologies, such as soil management techniques, low-cost planting materials, manual or semi-mechanized harvesting tools, post-harvest handling, and local knowledge and practices, presents a valuable opportunity within Brazilian agribusiness. This approach fosters a circular economy by connecting various sectors and contributing to regional sustainability [3].

Forage palm valorization can contribute to achieving Sustainable Development Goals by promoting sustainable agriculture, fostering economic growth, and mitigating climate change. These actions enhance food security, exemplifying the interconnectedness of environmental, social, and economic sustainability efforts. The Sustainable Development Goals, also known as the Global Goals, aim to preserve planetary habitability, eradicate poverty, and ensure peace and prosperity for all [4,5].

Furthermore, forage palm is a nutritionally valuable food source due to its high productivity and hardiness. The cladodes are rich in dietary fibers (pectin, lignin, cellulose, and hemicellulose) and abundant in antioxidants, such as ascorbic acid and phenolic compounds (hydroxybenzoic and hydroxycinnamic acids and flavonoids) [6]. This cactus has diverse industrial applications due to its unique composition. For example, pectin can be used as a thickening agent, and flavonoids show potential health benefits, exhibiting anti-inflammatory, antidiabetic, antiviral, and antioxidant properties; modulation of enzymatic activity, and inhibition of cell proliferation [7,8,9,10]. The vegetable can be consumed fresh or processed into various products, with flour production being particularly notable.

Vegetable flours, rich in dietary fiber and bioactive compounds, offer a clean-label approach to increasing fiber content in various food products, including bakery goods, pasta, soups, desserts, and dairy derivatives [11]. These flours contribute to the development of innovative and value-added food items. A critical step in flour production is drying, which reduces moisture content to below 15%. Common drying equipment includes convective dryers, circulating air ovens, air jet dryers, fluidized bed dryers, and microwave ovens. Selecting the optimal drying method is crucial for maintaining flour quality [12,13,14].

Microwave drying has emerged as a preferred method due to its superior efficiency compared to traditional drying techniques. This efficiency stems from the direct interaction of microwave energy with polar molecules, primarily water, within the material [15]. Water molecules in food are polar, having a positive electrical charge at one end and a negative charge at the other. When exposed to microwave radiation, these molecules align with the alternating electric field produced by the waves. The rapid alternation of the electric field causes the water molecules to vibrate intensely and rotate around their axes, generating heat through friction. This heat generated within the food leads to rapid evaporation of the water, resulting in effective drying [16].

Unlike conventional ovens, which heat the entire chamber, microwave drying focuses on heating the food directly, thereby minimizing the warming of the surrounding air. This approach leads to reduced energy consumption and shorter drying times. Microwave drying also offers selectivity, as it primarily targets the water molecules, thereby minimizing damage to other food components and better-preserving nutrients. The rapid and uniform heating process also inhibits microbial growth, enhancing food safety while preserving color and flavor. Additionally, the reduced drying time helps to retain heat-sensitive compounds [12,13,17].

Pasta remains a highly popular food choice due to its sensory appeal, nutritional value, convenience, and versatility. While pasta is a traditional staple, the market has seen continuous innovation with the introduction of whole grain, multigrain, gluten-free, and vegetable-enriched varieties [18]. Fortification of pasta with high-value ingredients is recognized as a crucial strategy for enhancing its nutritional profile globally. Organizations like the World Health Organization (WHO) and the Food and Drug Administration (FDA) actively promote such advancements [19].

The incorporation of fibers into pasta presents technological challenges, as adding dietary fibers reduces water availability for proper component hydration, potentially affecting product properties like hardness, chewiness, and volume [20]. However, the mucilage in palm polysaccharides can mitigate this issue by facilitating pasta hydration during the cooking process.

Therefore, this study aimed to obtain integral forage palm flour using microwave drying for blending with wheat flour in fresh and dry fettuccine-type pasta. The thermomechanical properties of the flours (blends) and the technological and sensory properties of the pasta were evaluated.

## 2. Materials and Methods

### 2.1. Materials

Forage palm cladodes were collected in the city of Couto Magalhães de Minas, Minas Gerais, Brazil (altitude 740 m, 18°04′16″ S and 43°28′31″ W). The project was registered under number AF2C488 in the National System for the Management of Genetic Heritage and Associated Traditional Knowledge (SisGen) of the Ministry of Environment of the Federative Republic of Brazil. Refined wheat flour (13% protein and 0.8% ash) was purchased from a local store in the city of Diamantina, Minas Gerais. All chemical reagents used to develop this work were of analytical grade and possessed the required purity per the chosen analytical methodology.

### 2.2. Methods

#### 2.2.1. Receiving, Sanitizing, and Processing Cladodes for Analysis and Flour Preparation

The cladodes of forage palm were initially cleaned with potable water to remove surface impurities. Spines were then manually removed using a knife. The cladodes were sanitized in a 200 ppm NaClO solution (*v*/*v*) for 15 min, followed by rinsing with potable water to eliminate residual chlorine. For centesimal composition analysis, the cladodes were crushed using a PMX-700 mixer (Philco, Curitiba, Brazil) to produce a puree. For the drying process, the cladodes were manually cut into cubes (approximately 94 × 72 × 110 mm, height × width × length). The samples were packaged into high-density polyethylene bags and then frozen in a DFN41 freezer (Electrolux, Curitiba, Brazil) at −18 °C until analysis.

#### 2.2.2. Proximate Composition of Integral Forage Palm Cladodes

The integral forage palm puree was characterized regarding its proximate composition, determined by the contents of moisture (934.01), protein (920.152; N = 6.25), lipids (920.39), ash (942.05), glucose as quantified by a colorimetric reaction using GOPOD (glucose oxidase peroxidase) (982.14), and total dietary fiber (978.10) [21]. The total reducing sugars expressed in glucose and non-reducing sugars expressed in sucrose were quantified by a colorimetric reaction using DNS (3,5-dinitro salicylic acid) as described by Santos et al. [22].

The elemental composition of minerals was determined using an EDX-7200 energy-dispersive X-ray fluorescence spectrometer (Shimadzu, Kyoto, Japan). Air-dried fine soil with a 40-mesh particle size was used as a matrix. Samples were prepared in 32 mm diameter by 23 mm height polyethylene sample holders covered with 6 µm Mylar^®^ film. EDXRF analysis was conducted under vacuum conditions using the following parameters: tube voltage of 15 keV (Na to Sc) and 50 keV (Ti to U), tube current of 184 and 25 μA, 10 mm collimator, 200-s real integration time, ambient temperature, and a liquid nitrogen-cooled Si(Li) detector.

#### 2.2.3. Microwave Radiation Drying Process

**Figure 1:**
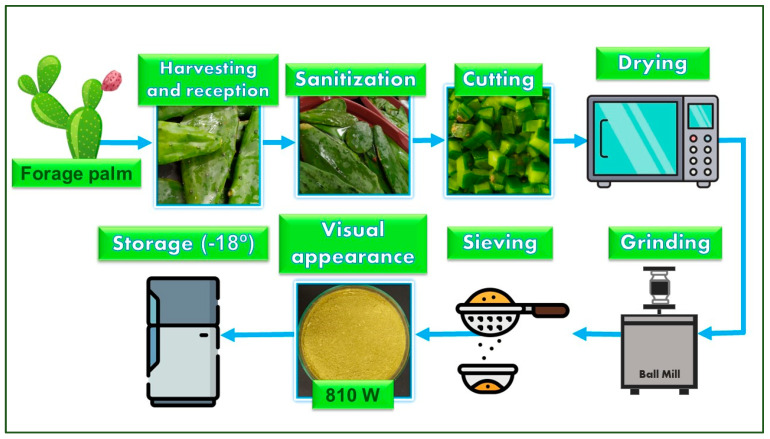
Flowchart for the microwave drying process of integral forage palm. Icons acquired from Flaticon^®^ available at: https://www.flaticon.com/, accessed on: 6 March 2024.

Integral forage palm cubes were weighed and subjected to microwave drying in an NN-GT672WRUN microwave oven (Panasonic, Manaus, Brazil) at 810 W for 10 min, with sample weighing every 30 s. Drying was carried out until the cubes reached a moisture content below 12%. After the drying process, the cubes were ground into flour in a TE-350 ball mill (Tecnal, Piracicaba, Brazil), sieved (250 µm opening), packaged in bioriented polypropylene bags, wrapped with aluminum foil for light protection, and stored under freezing conditions in a DFN41 freezer. The general flowchart for the preparation of the flours is presented in Figure 1.

#### 2.2.4. Instrumental Color of Integral Forage Palm Puree and Flour

Instrumental color was evaluated according to the CIELab* system, in triplicate, using a CM-5 Konica spectrophotometer (Minolta, Chiyoda, Japan), with the equipment configured with a D65 illuminant, 10° viewing angle, and calibration in RSIN mode (specular reflectance included).

#### 2.2.5. Total Soluble Phenolic Compounds of Integral Forage Palm Puree and Flour

The content of total soluble phenolic compounds was determined according to the method described by Pico et al. [23], with some modifications. Accurately weighed aliquots of puree (2.5 g) and flour (1 g) (dry basis) were subjected to extraction using 9 mL of a ternary methanol/water/acetone (0.17:0.50:0.33, *v*/*v/v*) solution in aluminum-foil wrapped conical Falcon tubes. The extraction process was conducted on an SL 180/DT shaker (Solab, Piracicaba, Brazil) at 240 rpm for 16 h, followed by centrifugation at 2500× *g* for 10 min using a Sorvall ST 8 centrifuge (Thermo Fisher Scientific, Waltham, MA, USA). The supernatant was quantitatively transferred to a 10 mL volumetric flask and filled to volume with the extraction solvent. To determine total soluble phenolic compounds, 100 µL of the phenolic extract was combined with 250 µL of 0.2 N Folin–Ciocalteu reagent, 3 mL of distilled water, and 1 mL of 15% Na_2_CO_3_ solution. The mixture was incubated in the dark for 30 min to allow color development. The 7-point standard curve was constructed using a gallic acid standard curve (0 to 600 mg·L^−1^; y = 0.0017x + 0.0079; r = 0.9931). Absorbance measurements were performed at 750 nm using a UV-M5 1 spectrophotometer (Bel Photonics, Monza, Italy). Each sample was analyzed in duplicate with four technical replicates. The total soluble phenolic compound content was expressed as mg of gallic acid equivalents per 100 g of sample (dry basis).

#### 2.2.6. Experimental Design for Fresh and Dry Extruded Fettuccine-Type Pasta

A completely randomized experimental design was employed for the formulation of the fettuccine-type pasta. The blends of refined wheat flour and integral forage palm flour were as follows: Control (100:00), P5 (95:05), P10 (90:10), P15 (85:15), and P20 (80:20) (*w*/*w*).

#### 2.2.7. Thermomechanical Properties of Flour Blends

The thermomechanical properties of the samples were analyzed using a Mixolab2^®^ (Chopin Technologies, Villeneuve-la-Garenne, France) following the method 54-60.01 of the American Association of Cereal Chemists International [24] and the Chopin^+^ protocol. A total of 75 g of sample was used for each analysis. The amount of water added to the dough was based on consistency to achieve an initial torque (C1) of 1.1 Nm. The analyses were performed in duplicate, and the following parameters were obtained: water absorption, stability, C1, C2, C3, C4, C5, C2-C1, C3-C2, C4-C3, C5-C4, slope-α, slope-β, and slope-γ [25].

#### 2.2.8. Water Absorption Index and Water Solubility Index

Water absorption index and water solubility index analyses were performed according to the method described by Schmiele et al. [26]. Briefly, Falcon tubes (15 mL) were weighed with lids, and 1 g (dry basis) of sample was added to each tube, followed by 10 mL of distilled water. The closed tubes were manually shaken intermittently every 5 min for 30 min. Subsequently, the samples were subjected to phase separation in a FANEM Baby|Centrifuge (Tecnal, Piracicaba, Brazil) for 10 min at 3200× *g*. The supernatant was collected in pre-weighed Petri dishes and the liquids were evaporated in a TE-394/1 oven (Tecnal, Piracicaba, Brazil) at 85 °C with circulation (1 m·s^−1^) and air renewal overnight. Then, air renewal was turned off, and the temperature was increased to 105 °C for 4 h for final dehydration. The analysis was performed in triplicate and the results were expressed in g of water per g of sample (dry basis) for the water absorption index and as a percentage (*w*/*w*) for the water solubility index (dry basis).

#### 2.2.9. Preparation of Fresh and Dried Extruded Fettuccine-Type Pasta

**Figure 2:**
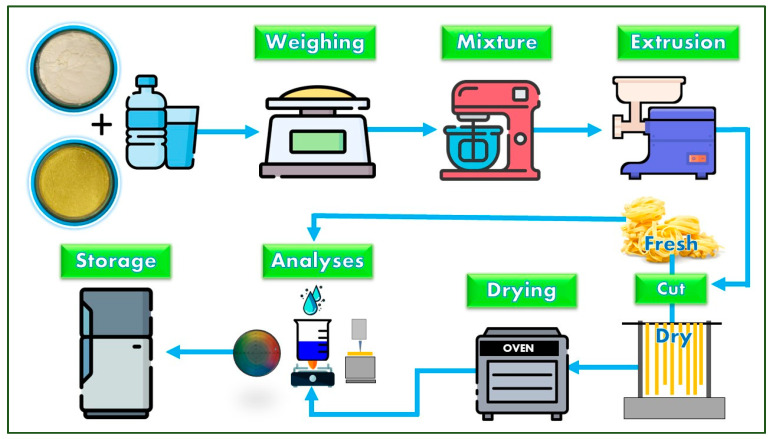
Flowchart of the processing of fresh and dry fettuccine-type pasta. Icons acquired from Flaticon^®^ available at: https://www.flaticon.com/, accessed on: 6 March 2024.

The formulations of the fettuccine-type pasta were Control (700 and 0 g), P5 (665 and 35 g), P10 (630 and 70 g), P15 (595 and 105 g), and P20 (560 and 140 g), respectively, for refined wheat flour and integral forage palm flour. The fettuccine-type pasta preparation process was carried out according to the methodology described by Schmiele et al. [26] and as shown in the flowchart in Figure 2. Water (quantum satis) was added to the control sample, and the amount of water required for the other formulations was calculated in direct proportion to the water hydration capacity of the blends. The mixing was performed in a Turbo PHP500 planetary mixer (Philco, Manaus, Brazil) slowly and continuously under agitation for 3 min, followed by a 7 min rest period for hydration equilibrium. The hydrated flour was subjected to conventional extrusion in an AELI-720 extruder (Braesi, Caxias do Sul, Brazil) in the fettuccine format, using a die with 32 openings with a 7.5 mm width and 2.5 mm thickness. Each batch was divided into two batches, one of which was evaluated as fresh fettuccine-type pasta and the other subjected to the drying process to obtain the dry fettuccine-type pasta.

Fresh fettuccine-type pasta was dehydrated to produce dry fettuccine-type pasta using a drying process (Figure 2). Pasta stands approximately 40 cm in length were suspended on stainless steel rods and dried in a TE-394/1 oven (Tecnal, Piracicaba, Brazil) at 40 °C for a total of 4.5 h. During the initial 2 h, air circulation (1 m·s^−1^) was maintained without renewal, and a water tray (0.02 m^2^ with 2 L water) was placed on the bottom of the oven. Subsequently, the water tray was removed, air renewal was initiated, and drying continued until the final moisture content of the product was below 14%. Both fresh and dehydrated fettuccine-type pasta were stored in biaxially oriented polypropylene packaging before analysis.

#### 2.2.10. Moisture Content and Cooking Characteristics of Fettuccine-Type Pasta

The moisture content of the fettuccine-type pasta samples was determined using the 44-15.02 method, and their cooking characteristics were evaluated following the 66-50.01 method [24]. To determine the optimal cooking time (min), 10 g samples were cooked in 140 mL of distilled water until they reached an al dente texture. The evaluated parameters were optimal cooking time (min), solid loss in cooking water (%, *w*/*w*), and weight gain (%, *w*/*w*), all determined in triplicate.

#### 2.2.11. Texture Characteristics of Fettuccine-Type Pasta

The texture profile of the fettuccine-type pasta was evaluated using a TAXT Plus texture analyzer (Micro Stable System, Haslemere, UK). The fracturability of dry fettuccine-type pasta, and the firmness, work of shear, stickiness, and elasticity of both fresh and cooked pasta (at an optimal cooking time) were determined. The fracturability of the dried fettuccine-type pasta was determined using a 3-Point Bending Rig probe (HDP/3PB). Ten replicate measurements were conducted on single fettuccine-type pasta strands, following the approach of Ungureanu et al. [27]. Test conditions included a test speed of 0.50 mm·s^−1^, a height of 20 mm, a distance of 5 mm in compression mode, and a force threshold of 0.049 N.

The 66-50.01 method [24] was employed for testing firmness, work of shear, and adhesiveness. Ten replicate measurements were conducted on six fettuccine-type pasta strands for each sample. A A/LKB-F probe was used with pre-test and test speeds of 0.50 mm·s^−1^, a post-test speed of 10.0 mm·s^−1^, a height of 15 mm, a distance of 14 mm, and a force threshold of 0.049 N. Pasta elasticity was assessed based on the methodology of Jaekel et al. [28]. The tensile force was applied to a single fettuccine-type pasta strand until rupture, with ten replications. The A/SPR probe test parameters included a test speed of 3.0 mm·s^−1^ and a distance of 70 mm.

#### 2.2.12. Sensory Analysis of Unflavored and Flavored (Garlic and Oil) Fettuccine-Type Pasta

Sensory analysis of fresh and dry cooked fettuccine-type pasta with 10% integral forage palm flour (based on favorable technological properties) was conducted by ethical guidelines approved by the Research Ethics Committee of the Federal University of Jequitinhonha and Mucuri Valleys (CAAE: 89302718.7.0000.5108). The samples were subjected to sensory evaluation by 85 untrained panelists. Samples were coded using a three-digit system and presented monadically in random order. Panelists utilized a 9-point hedonic scale (9—liked extremely, 1—disliked extremely) to assess overall acceptance and a 5-point purchase intention scale (5—would certainly buy, 1—would certainly not buy) [29]. To minimize carryover effects between samples, panelists rinsed their mouths with water. An acceptability index was calculated according to Equation (1).

Acceptability index (%) = Average score · 100/maximum score(1)

where the average score was given by the panelists regarding overall impression, and the maximum score is the maximum value of the hedonic scale.

One hundred grams of fettuccine-type pasta samples were cooked in twelve identical batches using 1.4 L of boiling water and 2.5% (*w*/*v*) iodized table salt. Cooking times were 8.61 min for fresh and 11.69 min for dry fettuccine-type pasta. To assess potential flavor enhancement, a subset of freshly cooked pasta (P10) was further prepared with extra virgin olive oil and fresh garlic, creating a “garlic and oil pasta” variant. These samples were portioned into 15 g servings, packaged in 50 mL biaxially oriented polyethylene containers, sealed, and frozen at −18 °C in a DFN41 freezer. For sensory evaluation, frozen samples were thawed and heated in an NN-GT672WRUN microwave oven (Panasonic, Manaus, Brazil) at 810 W for 2 min. The same panelists evaluated fresh-cooked and flavored fettuccine-type pasta. An open-ended question regarding potential sauce pairings was included in the sensory evaluation form.

#### 2.2.13. Statistical Analysis

The normality of the data was checked using the Shapiro–Wilk test. The differences in instrumental color and total soluble phenolic compounds of integral forage palm puree and flour were determined using the Student’s *t*-test (*p* < 0.05). Analyses conducted for flour blends (thermomechanical properties, water absorption index, water solubility index) and technological parameters (moisture, cooking, and texture characteristics) of fettuccine-type pasta were evaluated by one-way analysis of variance (*p* < 0.05), assuming a Gaussian normal distribution and homogeneity of variances. When significant differences were observed, the Tukey test (*p* < 0.05) was applied to detect differences among the samples. The sensory analysis data were divided into two groups: fresh pasta vs. dry pasta, and fresh garlic and oil pasta. The comparison between fresh and dry pasta was performed using the Student’s t-test (*p* < 0.05). The results of the fresh pasta samples with garlic and oil were presented as means and standard deviations.

## 3. Results and Discussion

### 3.1. Proximate Composition of Integral Forage Palm Puree

Forage palm cladodes are characterized by high water content and low acidity, resulting in a high susceptibility to fast microbial spoilage, hindering their fresh commercialization [30]. Drying and grinding mitigate these issues, facilitating storage and transportation [31].

As shown in Table 1, the high moisture content responsible for tissue turgor and visual appeal is influenced by environmental factors [32,33]. Protein content, a crucial indicator of cladode quality and nutritional value, was determined to be 1.88 ± 0.17% (wet basis) and significantly higher on a dry basis (15.80%). Ether extract, encompassing lipids, waxes, and some vitamins and pigments, was 0.14 ± <0.01%, while ash content was 1.80 ± 0.06% (wet basis) and 15.13% (dry basis). High ash content, often linked to soil salinity and mineral bioavailability [34], indicates abundant mineral content [35].

Non-reducing sugars (in sucrose), reducing sugars (in glucose), and glucose levels were 4.25, 63.47, and 2.49 mg·100 g^−1^, respectively. It is important to highlight that Lima et al. [35] did not find values of reducing and non-reducing sugars in the centesimal composition of the pulp of palm cladodes, noting that they collected shoots. This can be explained by Ribeiro et al. [36], who stated that older cladodes contain nearly twice the amount of sugars compared to younger cladodes. Dietary fiber comprises 67.30% of the flour (dry basis), reinforcing the forage palm’s status as a fiber-rich resource [37]. Its positive impact on intestinal health is well-established [38,39].

Eleven mineral elements were identified in integral forage palm. While sodium and magnesium were likely present, the equipment employed only quantified elements from aluminum to uranium, excluding these elements from the analysis. Calcium, the most abundant mineral in the human body, was the predominant element (65.03 ± 0.63%), essential for various bodily functions. Potassium (26.67 ± 0.42%) was also significant, a common soil nutrient and agricultural fertilizer known to enhance plant growth and resilience. Chlorine (7.30 ± 0.13%) was present in substantial amounts, while other elements, including sulfur (0.30 ± 0.13), strontium (0.26 ± <0.01), manganese (0.18 ± <0.01), silver (0.10 ± <0.01), rubidium (0.08 ± <0.01), iron (0.06 ± 0.01), zinc (0.04 ± <0.01), and copper (0.03 ± <0.01), were detected in lower concentrations.

### 3.2. Color Parameters and Total Soluble Phenolic Compounds in Integral Forage Palm Puree and Flour

Messina et al. [40] reported color as a critical determinant of food quality, often serving as the initial consumer evaluation criterion. The integral forage palm puree showed a dark greenish-yellow hue, and the integral forage palm flour had a lighter, reddish-yellow hue (Table 2). This chromatic variation is attributed to chlorophyll’s (*Chl*) light absorption properties in the blue, violet, and red wavelengths, with subsequent green light emission. Chlorophyll is composed of porphyrin and hydroporphyrin rings coordinated with a magnesium atom [41]. Chlorophyll *a* (*Chl a*) possesses a methyl group, while chlorophyll *b* (*Chl b*) has a carbonyl group at the primary carbon (aldehyde), rendering it more unstable due to its lower polarity and higher electronegativity. Heat treatment induces the loss of Mg^+2^ from chlorophyll molecules, transforming them into pheophytin *a* and pheophytin *b*, consequently resulting in a paler, olive-green hue [42]. Carotenoids typically accompany chlorophyll, protecting against oxidative stress and free radical sequestration. Carotenoids consist of carotenes (non-oxygenated) and xanthophylls (oxygenated), exhibiting non-polar characteristics and colors ranging from yellow to red [43].

Dick et al. [44] reported L*, a*, and b* color parameters of 68.83, −6.43, and 23.74, respectively, for *Opuntia monacantha* mucilage and flour, indicative of a pale green appearance. In contrast, our study yielded lower L* and higher a* and b* values. Messina et al. [40] reported significantly higher L* values (80.43 to 83.61), correlating with increased water content in the original raw material.

The total soluble phenolic compound content of whole forage palm flour was 30.27% lower than that of the pulp (Table 2), likely attributed to the drying process conditions. Di Bella et al. [37] reported that pruning season influences nutrient composition, with minerals and most phenolic acids and flavonoids exhibiting a higher abundance in summer. Compared to the values reported by Alves, Costant, and Teles [45] of 40.24 mg GAE·100 g^−1^ for fresh cactus and 4.86 mg GAE·100 g^−1^ for oven-dried flour, our study revealed significantly higher values for both pulp and microwave-dried flour. It is important to highlight that the (poly)phenolic compound content in *Opuntia ficus-indica* cladodes varies significantly depending on factors such as cladode age, part (peel or pulp), and cultivar [46].

### 3.3. Thermomechanical Characteristics of Pre-Mixes by Mixolab2

**Figure 3:**
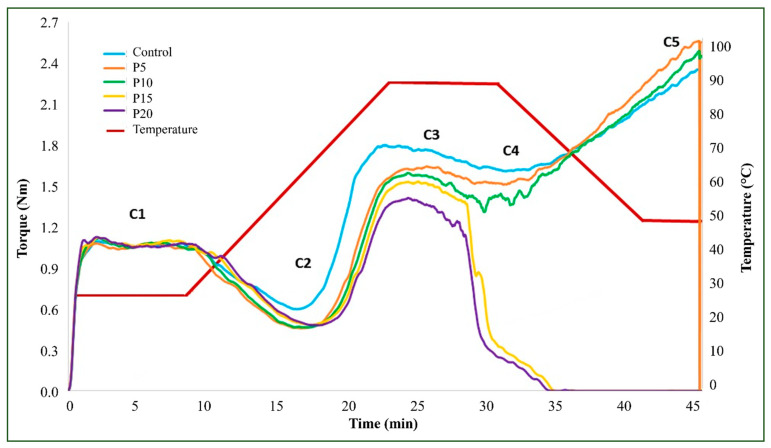
Thermomechanical curve and parameters analyzed for wheat flour mixed with integral forage palm flour at different levels: 0% (Control), 5% (P5), 10% (P10), 15% (P15), and 20% (P20). C1—maximum torque during mixing; C2—protein weakening based on mechanical work and temperature increase; C3—maximum torque during the heating stage, expressing the rate of starch gelatinization; C4—minimum torque during the heating period, indicating the stability of the hot gel formed; C5—torque after cooling at 50 °C, representing starch retrogradation during the cooling stage.

Table 3 presents the thermomechanical properties of wheat flour blended with varying proportions of integral forage palm flour, while Figure 3 visually depicts these trends. Samples containing 5, 10, and 15% integral forage palm flour (P5, P10, and P15) exhibited no significant differences in water absorption compared to the control. However, the P20 sample required water adjustment to attain the initial torque of 1.1 Nm. No significant variations were observed in dough stability or the C1 parameter (maximum initial consistency) among the samples. Conversely, the control sample exhibited a higher C2 parameter (minimum torque related to protein weakening) compared to the other samples. This suggests that the incorporation of integral forage palm flour weakens the gluten protein network through protein dilution, disrupting the starch-protein matrix, decreasing dough elasticity, and causing dough weakening during continued mixing [25,47].

The C3 parameter, representing maximum torque during the heating phase (associated with starch gelatinization), was highest for the control sample and decreased with increasing integral forage palm flour concentration. The P20 sample exhibited the lowest C3 value, indicating that substituting wheat flour with integral forage palm flour negatively impacts the dough gelatinization rate. The reduced starch availability caused by wheat flour dilution and the addition of water not fully absorbed by the medium contributed to decreased dough viscosity. The viscosity of cactus mucilage can vary during heating, with its thermal stability depending on the composition of the food system [48]. Regarding the C4 parameter (minimum torque during the heating phase, related to endogenous α-amylase activity and starch stability), only the P5 sample showed no difference from the control. Samples with 10%, 15%, and 20% integral forage palm flour (P10, P15, and P20) exhibited progressively lower C4 values.

Regarding the C5 parameter (torque after cooling to 50 °C, indicating starch retrogradation), samples containing 15% and 20% integral forage palm flour (P15 and P20) exhibited no torque. Conversely, samples with 5% and 10% integral forage palm flour (P5 and P10) showed higher C5 values compared to the control, suggesting a potential reduction in shelf life for solely wheat-based baked products due to increased water loss (syneresis or migration). However, this effect might be less detrimental in pasta production, in which starch recrystallization can enhance pasta firmness and cooking quality. Analyses of secondary dough parameters (C2-C1 and C3-C2) revealed higher values for the control sample compared to the P15 and P20 samples, which exhibited the lowest values for both parameters.

No significant differences were observed in the enzyme degradation rate (slope-*γ*) among samples. However, the protein weakening rate under heating (slope-*α*) varied significantly between P5 and P20, with P5 exhibiting the lowest and P20 the highest values. The starch gelatinization rate (slope-*β*) decreased with increasing integral forage palm flour content, with the most pronounced reduction observed in P20 compared to the control. The presence of mucilages and hydrocolloids reduces the hydration rate of wheat flour, thereby slowing down the gelatinization process [49].

The addition of integral forage palm flour enhanced dough handling during the mixing and extrusion stages of pasta production. However, the resulting gel structure exhibited instability under the influence of constant heat and agitation. Given the static conditions of pasta drying, further investigation is warranted to assess the potential impact of these findings on the final product.

### 3.4. Water Absorption Index and Water Solubility Index

**Figure 4:**
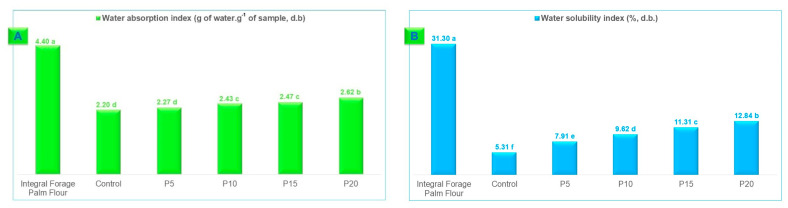
Water absorption index (**A**) and water solubility index (**B**) of wheat flour mixed with integral forage palm flour at different levels: 0% (Control), 5% (P5), 10% (P10), 15% (P15), and 20% (P20). Data are the means of three replicates. d.b = dry basis. Different lowercase letters above the bars denote statistical differences among the samples (ANOVA, Tukey test, *p* ≤ 0.05).

Integral forage palm flour exhibited significantly higher water absorption (4.40 g of water·g^−1^ of sample) and water solubility (31.30%) indices compared to wheat flour and wheat flour blends containing 5%, 10%, 15%, and 20% integral forage palm flour (Figure 4).

Water absorption and solubility are essential properties for bakery and pasta products and are directly related to the levels of water-insoluble proteins and the high hydration capacity of dietary fibers [45]. These properties result in high hydration, increased viscosity, and gel formation [12]. The dietary fiber fraction, particularly rich in mucilage, possesses a high water-binding capacity that can enhance dough formation and bakery product development [50]. Additionally, Alves, Constant, and Teles [45] highlight that dietary fibers in forage palm significantly contribute to the increase in the water absorption index, due to the hydrogen bonds formed by the functional groups (OH) of the biopolymers with water. Therefore, palm flour produced by microwave drying exhibits suitable characteristics regarding hydrodynamic properties, with significant values for both the water absorption and solubility indexes.

### 3.5. Technological Evaluation of Fettuccine-Type Pasta Quality

**Figure 5:**
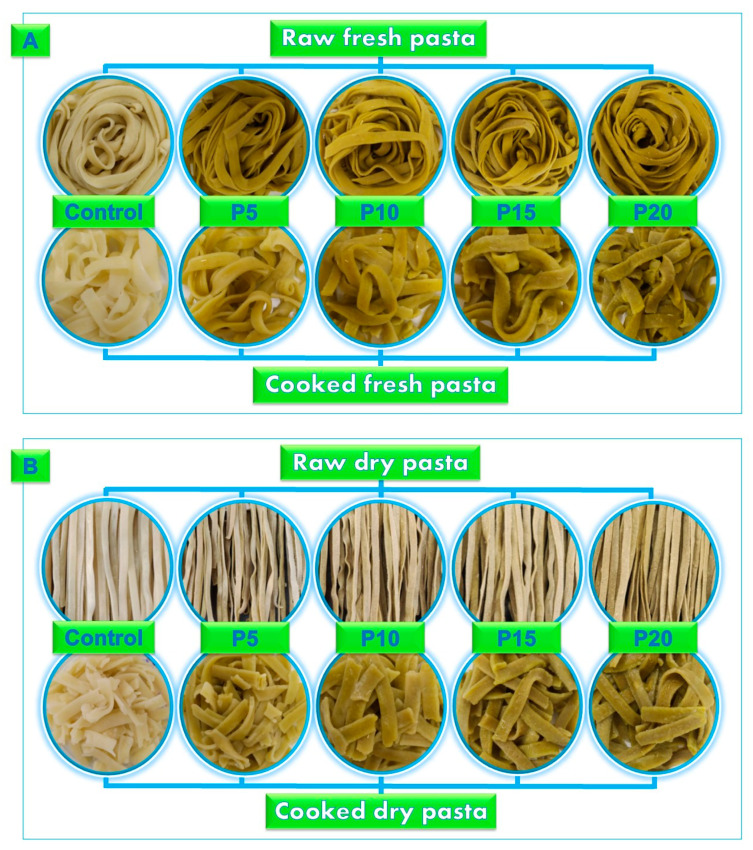
Visual appearance of fresh (**A**) and dried pasta (**B**) (raw and cooked). Wheat flour was replaced by integral forage palm flour at different levels: 0% (Control), 5% (P5), 10% (P10), 15% (P15), and 20% (P20).

Figure 5 presents visual comparisons of fresh raw and cooked (A), and dry raw and cooked (B) fettuccine-type pasta. As noted by Szydłowska-Tutaj, Złotek, and Combrzyński [51], consumers often select pasta based on its natural, uniform yellow color, which can vary depending on raw material and drying method.

#### 3.5.1. Moisture Content and Cooking Characteristics

High-quality pasta is characterized by flexibility, elasticity, and a non-sticky texture, with a pleasing flavor and aroma after cooking [49]. Additional quality attributes include a longer cooking time, minimal solid loss into cooking water (<6%), and a substantial weight increase (2–3 times) upon cooking [50]. The type of raw material, formulation, and processing techniques significantly influence pasta quality [51].

**Figure 6:**
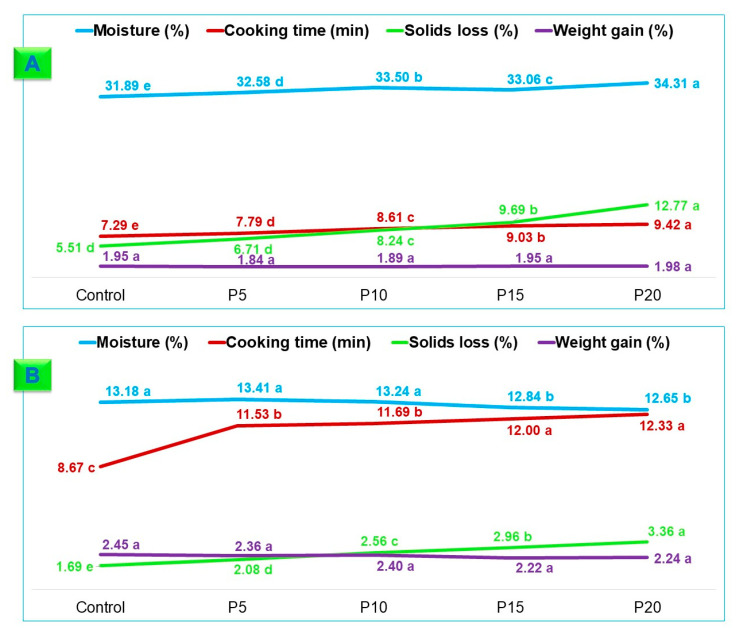
Cooking characteristics of fresh and dried fettuccine-type pasta. (**A**) Cooking characteristics of fresh pasta; (**B**) cooking characteristics of dry pasta. Wheat flour was replaced by integral forage palm flour at different levels: 0% (Control), 5% (P5), 10% (P10), 15% (P15), and 20% (P20). Data are the means of three replicates. Different lowercase letters at the same line denote statistical differences among the samples (ANOVA, Tukey test, *p* ≤ 0.05).

Fresh pasta is typically sold with a moisture content closely resembling its molded state to prevent strand adhesion. Figure 6A illustrates moisture content ranging from 31.89 to 34.31% in fresh fettuccine-type pasta samples, increasing with higher integral forage palm flour levels. While the two fresh fettuccine-type pasta formulations have significant differences, both fall within acceptable moisture parameters. It is essential to note that moisture content directly influences product stability, composition, storage, packaging, preparation, and overall quality [52].

Fresh fettuccine-type pasta exhibited satisfactory al dente textures according to the optimal cooking times. Cooking time increased as wheat flour was substituted with integral forage palm flour. Mucilage provides structural support to the dough by imparting viscoelasticity to the polymeric matrix surrounding the starch granules [53]. However, solid loss during cooking was higher with greater additions of whole palm forage flour, although no significant difference was found between the control and P5 samples (*p* > 0.05). Weight gain did not vary significantly among samples (*p* > 0.05), ranging from 1.84 to 1.98%.

Figure 6B illustrates that moisture content did not differ significantly between the control, P5, and P10 dry fettuccine-type pasta (*p* > 0.05), nor between P15 and P20 samples. For optimal storage and to prevent microbial and biochemical degradation, dried pasta should maintain a moisture content below 15% [54].

Similar to fresh fettuccine-type pasta, dried pasta cooking time increased with higher integral forage palm flour concentrations, ranging from 8.81 to 12.33 min. These values align with those of Ferreira et al. [55], who reported optimal cooking times of 12.5 to 13.0 min for durum wheat semolina pasta with bamboo fiber. It is crucial to note the difference in base flour (*durum* vs. *T. aestivum*) and added fiber (bamboo vs. forage palm).

Figure 6B shows increased solid loss in cooking water with more integral forage palm flour added to dry fettuccine-type pasta. Nevertheless, the drying process enhanced the pasta structure, leading to a lower solid loss compared to fresh fettuccine-type pasta. Palm mucilage in refined wheat flour outperformed bamboo fiber (5.01–9.14%) and cowpea/rice flours (4.39–6.17%) [56] in durum wheat semolina pasta regarding solid loss in the cooking water.

The weight increase of cooked dried fettuccine-type pasta remained unaffected by the integral forage palm flour content, possibly due to a balanced ratio of soluble and insoluble dietary fiber components. This is supported by the flour’s high water solubility (~30%, Figure 5) and aligns with Di Bella et al.’s [37] findings.

The balanced ratio of soluble and insoluble dietary fiber contributed to the substantial weight gain of the fettuccine-type pasta, and was more than double its initial weight across all trials (Figure 6). This result aligns with that of Ferreira et al. [55], who reported similar weight gain (2.21–2.80 times) for durum wheat semolina pasta with added bamboo fiber. Bruneel et al. [57] stated that a 200% weight increase is essential for optimal pasta water absorption and consumer satisfaction.

While many studies have explored pasta from unconventional sources with higher fiber and protein content, often leading to reduced weight and volume [56,57,58,59,60,61], the P10 pasta exhibited exceptional weight gain. This observation supports the retrogradation hypothesis indicated by the elevated C5 value in the thermomechanical analysis (Figure 3).

#### 3.5.2. Texture Characteristics

**Figure 7:**
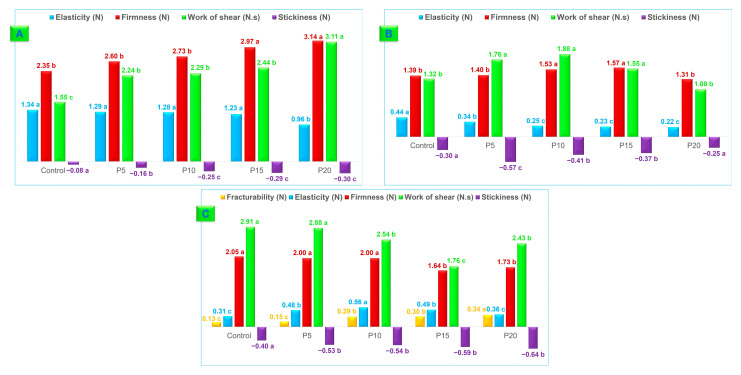
Texture characteristics of fresh, dried and cooked fettuccine-type pasta. (**A**) Fresh raw pasta; (**B**) cooked fresh pasta; (**C**) raw and cooked dry pasta. Wheat flour was replaced by integral forage palm flour at different levels: 0% (Control), 5% (P5), 10% (P10), 15% (P15), and 20% (P20). Data are the means of ten replicates. Different lowercase letters above or under the bars denote statistical differences among the samples (ANOVA, Tukey test, *p* ≤ 0.05).

The results of the texture analyses of fettuccine-type pasta are presented in Figure 7. Fresh raw fettuccine-type pasta elasticity decreased with increasing integral forage palm flour content. However, firmness, work of shear, and stickiness were higher than those of the control. While P5, P10, and P15 fresh pasta elasticity did not differ significantly from that of the control (*p* > 0.05), P20 elasticity was lower (0.96 N vs. 1.34 N Control). Cooking significantly reduced the elasticity of fresh fettuccine-type pasta in all samples, ranging from 0.44 N (Control) to 0.22 N (P20). The increase in pasta hardness when adding palm flour is related to the hydration capacity of the mucilage and the complex formed between the mucilage, proteins, and starch [62].

Integral forage palm flour increased fettuccine-type fresh pasta firmness, ranging from 2.35 N (Control) to 3.14 N (P20). While P5 firmness did not differ significantly from the control, all other samples showed increased firmness. Work of shear and stickiness also increased with the palm flour addition. Work of shear did not vary significantly among P5, P10, and P15 fettuccine-type fresh pasta. Stickiness ranged from −0.08 to −0.30 N, with no significant differences between P10, P15, and P20. Lower stickiness is desirable for pasta.

Firmness, work of shear, and stickiness of cooked fresh fettuccine-type pasta initially increased with up to a 10% integral forage palm substitution, then declined. This trend likely reflects the interplay between gluten network integrity and the influence of palm flour mucilage. Lower substitutions preserved the gluten structure, while higher levels disrupted it. Moreover, initial mucilage-gluten interactions might have enhanced texture.

Firmness increased for P10 and P15 fettuccine-type fresh-cooked pasta compared to the control, while P5 and P20 showed no significant differences. Work of shear peaked at P10 (1.88 N.s) after increasing from the control value (1.32 N.s), and subsequently declined. It is important to emphasize that for pasta to be of high quality, it should have high elasticity, firmness, and work of shear, and low stickiness. The increased force required to fracture fettuccine indicates enhanced pasta integrity. Palm mucilage likely contributed to this improvement during the drying process.

The results for fracturability (raw dry pasta) and elasticity, firmness, work of shear, and stickiness (cooked dry pasta) indicate that increasing the percentage of integral forage palm flour in fettuccine-type pasta correlates with a higher mechanical work requirement to induce fracture. The control sample showed similar results to P5, and the P10 and P15 samples were also statistically equal to each other. However, all samples were inferior to P20 in terms of fracturability.

It is noteworthy that the firmness values reported by Ferreira et al. [55], ranging from 9.70 to 16.70 N, were higher than those observed in this study. These authors utilized a partial substitution of *Triticum durum* semolina with bamboo fiber and young bamboo culm flour in their pasta. In comparison, the work of shear values were 26.63 to 29.87 N.s and 5.87 to 7.30 N.s, respectively.

A similar pattern was observed for stickiness, in which an increase in integral forage palm flour content resulted in greater adhesiveness in the pasta, likely due to the mucilage from the forage palm. The interaction of some hydrocolloids with starch or protein in noodles limits starch gelatinization, increasing the amount of soluble substances on the noodle surface [63,64]. This remark was confirmed by the increase in leached solids during the cooking process of the noodles. Overall, in line with the cooking parameter, the P10 fettuccine-type pasta demonstrated superior performance in instrumental texture analysis.

### 3.6. Sensory Evaluation of Fettuccine-Type Pasta Quality

Technological and nutritional properties, combined with functional and health-related properties, should be considered in the sensory evaluation of a food product. Regarding the technological characteristics, the fresh and dry fettuccine-type pasta samples labeled P10 stood out in various parameters. Fresh pasta required 8.61 min to cook, with a 1.89% weight gain and 8.24% solid loss in cooking water. Dry pasta took 11.69 min, gaining 2.40% in weight and losing 2.56% of solids. Fresh raw pasta exhibited excellent elasticity (1.28 N), firmness (2.73 N), high work of shear (2.29 N.s), and low stickiness (−0.25 N). After cooking, elasticity decreased to an intermediate level (0.25 N), while firmness remained high (1.53 N), work of shear increased (1.88 N.s), and stickiness remained reasonable (−0.41 N). Raw dry pasta demonstrated good fracture resistance (0.27 N). Cooked dry pasta showed increased elasticity (0.56 N), excellent firmness (2.00 N, comparable to the highest value of 2.05 N), high work of shear (2.54 N.s), and adequate stickiness (−0.54 N). Due to these outstanding results, P10 pasta was selected for sensory analysis.

#### 3.6.1. Cooked Fresh and Dry Fettuccine-Type Pasta

According to Table 4, it can be observed that only the texture parameter showed a significant difference between the fresh and dry pasta samples according to the Student’s t-test. Regarding the other parameters, the samples exhibited the same sensory acceptance by the recruited panelists. Aroma, appearance, and color were rated between indifferent and slightly liked. Taste and overall impression were rated close to slightly liked. Purchase intention indicated indifference among the panelists.

The texture of fresh fettuccine-type pasta received the highest score (6.88), indicating it was moderately liked. In contrast, dry fettuccine-type pasta was scored as slightly liked (6.31). Sensory analysis showed that fresh fettuccine-type pasta performed better, with an acceptability index of 69.44%. Consequently, it was selected for evaluation as a frozen, ready-to-eat option. Garlic and oil (extra virgin olive oil) were chosen for preparation, as their flavor does not significantly alter the product appearance, especially by not masking the final product’s color.

#### 3.6.2. Ready-to-Eat Garlic and Oil Fettuccine-Type Pasta

The samples were evaluated based on seven parameters, resulting in an aroma rated as “liked very much” (7.88 ± 1.07), appearance as “slightly liked” (5.90 ± 1.74), color as “slightly liked” (5.70 ± 1.63), texture as “moderately liked” (6.80 ± 1.68), flavor between “moderately liked” and “liked very much” (7.45 ± 1.56), overall impression as “moderately liked” (7.17 ± 1.44), and purchase intent on a scale of 0 to 5 of 3.89 ± 0.94. Fresh pasta flavored with garlic and extra virgin olive oil achieved a sensory acceptance rate of 79.67%.

With the proposal to explore the application of frozen garlic and oil pasta, panelists consumed the samples in their respective containers. They generally indicated a preference for consuming pasta in different forms. According to the transcribed results, 29.41% would use white sauce, 27.06% four cheese, 20% Bolognese, 18.82% chicken, and 4.71% tomato sauce. Consequently, the panelist suggested new ideas to enhance the product.

## 4. Conclusions

Integral forage palm flour demonstrated exceptional nutritional, functional, and technological properties, making it a promising ingredient for innovative food products, particularly bakery goods and pasta. Both fresh and dry fettuccine-type pasta formulations exhibited excellent cooking and texture performance, with P10 (integral forage palm flour replacing 10% wheat flour) standing out as the optimal choice due to its cooking time, solid loss, and weight gain. Fresh raw P10 pasta showed superior elasticity, firmness, and work of shear while maintaining low stickiness. After cooking, it retained high firmness and increased work of shear with acceptable stickiness. Dry P10 pasta offered good fracture resistance and, when cooked, demonstrated enhanced elasticity and sustained firmness, comparable to that of fresh pasta. Panelists discerned textural differences among pasta samples, favoring fresh pasta. Both pasta types achieved acceptance indices slightly below 70%, potentially attributed to the novelty of integral forage palm flour pasta. However, the introduction of garlic and extra virgin olive oil to fresh pasta (garlic oil pasta) significantly elevated acceptance to nearly 80%, highlighting the potential of strategic food processing techniques to promote unconventional ingredients.

## Figures and Tables

**Table 1:** Proximate composition of integral forage palm puree and flour obtained by a microwave drying process.

Component	Integral Forage Palm Puree (Wet Basis)	Integral Forage Palm Puree (Dry Basis)
Moisture (%)	92.04 ± 1.82	-
Proteins (%)	1.88 ± 0.17	15.80
Lipids (%)	0.14 ± <0.01	1.18
Ashes (%)	1.80 ± 0.06	15.13
Non-reducing sugars (mg of sucrose·100 g^−1^)	4.25 ± 4.06	35.71
Reducing sugars (mg of glucose·100 g^−1^)	63.47 ± 3.51	533.36
Glucose (mg·100 g^−1^)	2.49 ± 0.34	20.92
Total dietary fiber (%) *	4.08 ± 5.39	67.30

Data are the means ± standard deviation of three replicates (n = 3). * Total dietary fiber content calculated by the difference [100 − (proteins + ether extract + ashes + digestible carbohydrates)].

**Table 2:** Color parameters and total soluble phenolic compounds of integral forage palm puree and flour obtained by a microwave drying process.

Other Parameters	Integral Forage Palm Puree	Integral Forage Palm Flour
L*	42.14 ± 0.09	60.18 ± 0.05
a*	−5.57 ± 0.01	3.32 ± 0.04
b*	29.09 ± 0.12	27.46 ± 0.01
Total soluble phenolic compounds (mg GAE·100 g^−1^, d.b)	359.70 ± 13.55	250.81 ± 8.86

Data are the means ± standard deviation of three replicates (n = 3). GAE = gallic acid equivalent.

**Table 3:** Thermomechanical parameters of dough obtained by Mixolab2 of wheat flour mixed with integral forage palm flour at different levels: 0% (Control), 5% (P5), 10% (P10), 15% (P15), and 20% (P20).

Sample	Control	P5	P10	P15	P20
Water absorption (%)	58.00	58.00	58.00	58.00	59.7
Stability (min)	9.40 ± 0.42 ^ns^	8.85 ± 0.21 ^ns^	9.10 ± 0.14 ^ns^	9.75 ± 0.07 ^ns^	9.75 ± 0.64 ^ns^
C1 (Nm)	1.097 ± 0.006 ^ns^	1.091 ± 0.040 ^ns^	1.123 ± 0.007 ^ns^	1.128 ± 0.018 ^ns^	1.129 ± 0.005 ^ns^
C2 (Nm)	0.592 ± 0.001 ^a^	0.457 ± 0.016 ^b^	0.464 ± 0.004 ^b^	0.484 ± 0.013 ^b^	0.480 ± 0.013 ^b^
C3 (Nm)	1.811 ± 0.002 ^a^	1.648 ± 0.031 ^b^	1.598 ± 0.010 ^bc^	1.537 ± 0.024 ^c^	1.415 ± 0.024 ^d^
C4 (Nm)	1.595 ± 0.028 ^a^	1.506 ± 0.031 ^a^	1.278 ± 0.081 ^b^	0.323 ± 0.023 ^c^	0.312 ± 0.001 ^c^
C5 (Nm)	2.361 ± 0.015 ^c^	2.564 ± 0.015 ^a^	2.489 ± 0.011 ^b^	nd	nd
Slope-*α*	−0.072 ± 0.023 ^ab^	−0.125 ± 0.007 ^b^	−0.086 ± 0.025 ^ab^	−0.065 ± 0.007 ^ab^	−0.031 ± 0.0267 ^a^
Slope-*β*	0.454 ± 0.040 ^a^	0.296 ± 0.014 ^b^	0.288 ± 0.014 ^b^	0.282 ± 0.090 ^ab^	0.228 ± 0.059 ^b^
Slope-*γ*	−0.056 ± 0.025 ^ns^	−0.056 ± 0.020 ^ns^	−0.013 ± 0.024 ^ns^	nd	nd
C2-C1 (Nm)	−0.505 ± 0.007 ^a^	−0.634 ± 0.024 ^b^	−0.659 ± 0.011 ^b^	−0.644 ± 0.006 ^b^	−0.649 ± 0.018 ^b^
C3-C2 (Nm)	1.219 ± 0.003 ^a^	1.190 ± 0.015 ^a^	1.134 ± 0.006 ^b^	1.053 ± 0.011 ^c^	0.934 ± 0.011 ^d^
C4-C3 (Nm)	−0.216 ± 0.030 ^ab^	−0.142 ± 0.001 ^a^	−0.320 ± 0.090 ^b^	nd	nd
C5-C4 (Nm)	0.765 ± 0.012 ^c^	1.058 ± 0.016 ^b^	1.211 ± 0.070 ^a^	nd	nd

Data are the means ± standard deviation of three replicates (n = 3). Ns = not significant. Nd = not detected. Different lowercase letters in the row denote statistical differences among the samples (ANOVA, Tukey test, *p* ≤ 0.05).

**Table 4:** Sensory parameters of cooked fresh and dry pasta.

Attributes	Fresh Pasta	Dry Pasta
Aroma ^ns^	5.74 ± 1.72	5.85 ± 1.74
Appearance ^ns^	5.75 ± 1.77	5.54 ± 1.67
Color ^ns^	5.82 ± 1.70	5.62 ± 1.65
Texture	6.88 ± 1.71 *	6.31 ± 1.90 *
Taste ^ns^	6.16 ± 1.96	5.78 ± 1.94
Overall impression ^ns^	6.25 ± 1.75	5.96 ± 1.62
Purchase intention ^ns^	3.28 ± 1.09	3.09 ± 0.98
Acceptability index (%)	69.44	66.22

Data are the means ± standard deviation (n = 85). ns = not significant. * significant difference was observed between the samples by the Student’s *t*-test (*p* < 0.05).

## Data Availability

The original contributions presented in the study are included in the article, further inquiries can be directed to the corresponding author.
